# Whole-Transcriptome Analysis on the Leaves of *Rosa chinensis Jacq.* under Exposure to Polycyclic Aromatic Hydrocarbons

**DOI:** 10.3390/toxics11070610

**Published:** 2023-07-13

**Authors:** Shili Tian, Qingyang Liu, Jingming Qu, Ming Yang, Qiaoyun Ma, Jia Liu, Peng Shao, Yanju Liu

**Affiliations:** 1 Beijing Center for Physical and Chemical Analysis, Institute of Analysis and Testing, Beijing Academy of Science and Technology, Beijing 100089, China; tianshili@bcpca.ac.cn (S.T.); qujingming@bcpca.ac.cn (J.Q.); hotyang10@163.com (M.Y.); mqy08250825@163.com (Q.M.); jialiu@alumni.tongji.edu.cn (J.L.); 2 College of Biology and the Environment, Nanjing Forestry University, Nanjing 210037, China; qyliu@njfu.edu.cn

**Keywords:** PAHs, whole-transcriptome analysis, exposure, gene transcription levels

## Abstract

The leaves of plants can be recommended as a cheap and sustainable environmental protection tool to mitigate PAHs with high toxicity in the ambient environment because they can serve as a reactor to remove ambient PAHs. Although previous studies have demonstrated that PAHs exhibit toxicological features, our knowledge about how ambient PAHs influence the leaves of plants is limited regarding the leaves of plants reducing ambient PAHs as a reactor. In this study, 1-year-old *Rosa chinensis Jacq*. with good growth potential was selected as a model plant. The leaves of *Rosa chinensis Jacq*. were exposed to 16 types of PAHs in the environmental concentration exposure group (0.1 μg L^−1^) and high-concentration exposure group (5 μg L^−1^) for seven days. In comparison, the leaves of *Rosa chinensis Jacq*. were exposed to de-ionized water and were chosen as the control group. During the exposure periods, the physiological parameters of leaves including, chlorophyll value, water content, temperature and nitrogen, were monitored using a chlorophyll meter. After 7 days of exposure, the leaves in the control and exposure groups were collected and used for whole-transcriptome analysis. Our results demonstrate that significant differentially expressed genes were observed in the leaves of *Rosa chinensis Jacq*. exposed to individual PAHs at 5 μg L^−1^ compared to the control group. These differentially expressed genes were involved in seven main pathways using bioinformatic analyses. In contrast, the levels of PAHs at environmentally relevant concentrations had negligible impacts on the physiological parameters and the gene transcription levels of the leaves of *Rosa chinensis Jacq*. Our results may provide direct evidence to remove ambient PAHs using terrestrial trees without considering the risk of PAHs at environmentally relevant concentrations on the leaves of terrestrial plants.

## 1. Introduction

Polycyclic aromatic hydrocarbons (PAHs) are recognized as teratogenic, carcinogenic and mutagenic traits by the World Health Organization (WHO) [1,2]. PAHs originate from the incomplete combustion of fossil fuel and hydrogen-containing substances, including wood, crop straw, tobacco, etc. Most PAHs are emitted from mobile sources, biomass burning and coal combustion [3,4,5]. The emissions of PAHs in China account for ~20% of the total PAH emissions globally [6]. In North China, mean levels of particulate BaP (a representative PAH) vary from 1.1 to 14.3 ng m^−3^ annually, which is higher than the recommended threshold value of 1.0 ng m^−3^ by the WHO [7,8]. The high BaP concentration in particle matter could pose a health risk to public health and exhibit ecological toxicological effects on the ecosystem [9,10]. It is estimated that the inhalation of particulate BaP ranging from 1.1 to 14.3 ng m^−3^ could lead to an incremental lifetime cancer risk (ILCR) as high as 3.1 × 10^−5^, greater than the recommended safe level (10^−6^) [8].

The ecological remediation strategy is recommended as a cheap and sustainable environmental protection tool for mitigating PAHs with high toxicity in the ambient environment [11,12,13]. Trees could serve as filters for PAHs, and the leaves of plants could be treated as a reactor to remove ambient PAHs [14,15,16]. A previous study indicated that trees can remove approximately 710,000 tons of air pollutants, including NO_2_, SO_2_ and air pollutants, in particulate matter in 55 cities in the United States [17,18,19]. It is estimated that plant leaves can absorb 1.71 ± 0.05 g m^−2^ of air pollutants in particulate matter per week, from 1960 to 2016 based on 150 field studies across 15 countries [20,21]. Klingberg et al. [12] studied the PAH accumulation in *Quercus palustris* and *Pinus nigra* in the urban landscape of Gothenburg, Sweden. They found a strong association between gaseous PAH concentrations in leaves and the air. The concentrations of PAH were observed to be higher in 3-year-old black pine needles relative to those in 1-year-old black pine needles. In oak leaves, a significant decrease in the concentration of low-molecular-mass PAHs was found between June and September. In contrast, there was a significant increase in high-molecular-mass PAHs from June to September. Once PAHs are released into the atmosphere, they can reach out to the surfaces of leaves in the form of gas and in a particulate state [22]. The waxes of leaves are considered to be essential substances for ambient PAH enrichment [23,24]. Yang et al. [22] demonstrated the relationship between foliage uptake and the inner-leaf translocation of PAHs by *Cinnamomum camphora*. They found a negative correlation between the wax contents and the total concentration of 16 PAHs. The transportation of PAHs from foliar dust to cuticular wax was the primary pathway of leaf accumulation. The values of the translocation factor for PAHs from foliar dust to cuticular wax were observed to be highly dependent on an increasing tendency of low-molecular-weight PAHs and a decreasing tendency of high-molecular-weight PAHs. Tian et al. [24] assessed the differences in the uptake and accumulation of PAHs by leaves across eight plants in Shanghai, China. They showed the differences in the ability to uptake and absorb PAHs across eight plants due to variations in the morphology and physiological characteristics of leaves. The retention of low-molecular-weight PAHs in eight plant leaves was associated with leaf morphology and physiological characteristics, including surface roughness, stomatal density, polar components, etc. For medium- and high-molecular-weight PAHs, wax content and adsorption were found to be the dominant factors in the accumulation ability of eight plants.

Most PAHs can enter plant leaves through the stomata [23]. Lipid components in the waxes of leaves can interact with ambient PAHs through adsorption [25]. Then, the absorbed PAHs can penetrate the surfaces of leaves through the stomata and migrate to the internal tissues of the leaves [23]. Prigioniero et al. [25] used optical microscopy and infrared spectroscopy to explore the relationships between the uptake rates of PAHs and leaf surface functional traits in four Mediterranean evergreen trees, including *Chamaerops humilis*, *Citrus × aurantium*, *Magnolia grandiflora* and *Quercus ilex* during adry month. They suggested that cutin in the dewaxed leaves of evergreen trees is the main contributor to the uptake of PAHs.

Prior studies have documented the mitigation effects of PAHs by trees and the processes of PAHs from the ambient environment to the internal tissues of leaves [26,27,28]. De Nicola et al. [27] compared the ability to capture PAHs from ambient air between evergreen (*P. pinaster*) and deciduous trees (*Q. robur*). The results of the findings indicated that the uptake of PAHs increases with the diameter at the breast height of trees. The evergreen tree (*P. pinaster*) enhanced the ability to capture higher amounts of low- and medium-molecular-weight PAHs compared to that of the deciduous tree (*Q. robur*). Anna et al. [28] linked the ability to remove PAHs and the canopy water storage capacity of coniferous trees. They measured 18 types of PAHs in the needles of three coniferous trees, including *Pinus sylvestris* L., *Picea abies* (L.) *H. Karst* and *Abies alba mill.* Samples were collected in three locations in Czarna Rózga Forest Reserve, Poland. They found that increases in the canopy water storage capacity were associated with the total content of hydrophobic PAHs in needles within the same species. This study demonstrated that the canopy water storage capacity is an essential parameter for capturing hydrophobic PAHs.

However, limited studies have illustrated the toxicological effects of PAHs on plant leaves during the migrating processes from the ambient environment to the internal tissues [21]. Thus, this study aims to investigate the responses of plant leaves under exposure to PAHs at an environmentally relevant level and higher level withwhole-transcriptome analysis. This study could accumulate evidence on the physiological and pathological responses of plants under exposure to ambient PAHs.

## 2. Experimental Section

### 2.1. Exposure Experiments

In this experiment, 1-year-old *Rosa chinensis Jacq*. with good growth potential and no diseases were selected. Each individual of *Rosa chinensis Jacq*. was planted in a 15 cm-diameter flowerpot with a height of 15 cm. The cultivation environment had a photoperiod of 12/12 h (light/darkness). The exposure experiments included three groups, which were the control group, the low-concentration exposure group and the high-concentration exposure group. The exposure chemical reagents were a mixture of 16 polycyclic aromatic hydrocarbons [13]. The 16 types of PAHs were naphthalene, anthracene, acenaphthene, fluorene, phenanthrene, anthracene, fluoranthene, pyrene, benzo (a) anthracene, benzo (b) fluoranthene, benzo (k) fluoranthene, picene, indeno (1, 2, 3-cd) pyrene, benzo (g, h, i)perylene, dibenzo (a, i)pyrene and dibenzo (a, h)pyrene. We used the mixtures of 16 PAHs as the exposure chemicals because these 16 types of PAHs are frequently detected in the ambient environment [2,14,15,16]. The choice of the mixtures of PAHs rather than individual PAHs with high toxicity in the exposure experiment was in accord with the real environmental conditions [11,12,13]. An amount of 200 μg mL^−1^ of PAH mixture dissolved in a methanol solution was selected as the standard solution. The levels of the low-concentration exposure group and high-concentration exposure group were set to be 0.1 and 5 μg L^−1^ for individual PAHs, respectively. The level of 5 μg L^−1^ for individual PAHs was prepared using 100 μL of PAHs at 200 μg mL^−1^ and 4000 mL of de-ionized water. Then, the level of 0.1 μg L^−1^ for individual PAHs was prepared using 1 mL of PAHs at 5 μg mL^−1^ and 50 mL of de-ionized water. Thus, due to methanol being used as the solvent in the standard solution, it was estimated that the levels of methanol were 25 μL L^−1^ in the low-concentration exposure group and 0.5 μL L^−1^ in the high-concentration exposure group. The production of methanol in leaves occurred with leaf development and expansion [29]. It was found that the methanol emission of leaves ranged from 10.0 to 26.8 μg g^−1^ [29]. Because methanol existed in the leaves, it was expected that trace amounts of methanol existed in the exposure solution, ranging from 0.5 to 25 μL L^−1^, which have negligible effects on the physiological conditions of the leaves. Each group included four replicates.

Before the exposure experiment, the leaves of *Rosa chinensis Jacq.* were washed with de-ionized water three times. Then, in the low-concentration exposure group, the levels of individual PAHs at 0.1 μg L^−1^ were sprayed on the leaves of *Rosa chinensis Jacq.* once a day, and in the high-concentration exposure group, 5 μg L^−1^ of individual PAHs was sprayed on the leaves once per day. The control group was carried out with the same procedure using de-ionized water on four replicated healthy leaves with similar ages and leaf areas [30]. During the experiment, the levels of chlorophyll, leaf water content and nitrogen in the leaves were measured using a chlorophyll meter (YT-YC, YunTang Technology, Shandong, China) simultaneously every day [31,32]. For each measurement, four leaf replicates in each group were measured. The mean levels of chlorophyll, leaf water content, and nitrogen in the leaves were treated as the daily mean concentrations.Prior studies have shown that the overall degradation half-lives of PAHs on leaves varies from approximately 0.6 to 7 days at room temperature [33]. It is expected that the trace levels of PAHs are decomposed due to the photo- and biodegradation effect within one week. To conduct the exposure experiment in real environmental conditions, the entire exposure experiment was preceded by 7 days at a temperature ranging from 18 to 20 °C and humidity ranging from 40 to 50%. After the exposure experiment, 5 g of leaves of each sample from the low-concentration exposure group and high-concentration exposure group was collected and stored at −80 °C for transcriptomic sequencing analysis.

### 2.2. Transcriptome Sequencing

RNA quantification and the qualification of RNA integrity in leaves were assessed using the RNA Nano 6000 Assay Kit of the Bioanalyzer 2100 system (Agilent Technologies, Santa Clara, CA, USA) [34]. Total RNA was used as input material for the RNA sample preparations. Briefly, mRNA was purified from total RNA using poly-T oligo-attached magnetic beads. Fragmentation was carried out using divalent cations under an elevated temperature in First-Strand Synthesis Reaction Buffer (5X). First-strand cDNA was synthesized using a random hexamer primer and M-MuLV Reverse Transcriptase (RNase H-). Second-strand cDNA synthesis was subsequently performed using DNA Polymerase I and RNase H. Remaining overhangs were converted into blunt ends via exonuclease/polymerase activities. After the adenylation of the 3′ends of DNA fragments, adaptors with hairpin loop structures were ligated to prepare for hybridization. To select cDNA fragments that were preferentially 370~420 bp in length, the library fragments were purified with the AMPure XP system (Beckman Coulter, Beverly, MA, USA). Then, PCR was performed with Phusion High-Fidelity DNA polymerase, universal PCR primers and index (X) primer. Last, PCR products were purified (AMPure XP system), and library quality was assessed on the Agilent Bioanalyzer 2100 system. The clustering of the index-coded samples was performed on a cBot Cluster Generation System using TruSeq PE Cluster Kit v3-cBot-HS (Illumia, San Diego, CA, USA) according to the manufacturer’s instructions. After cluster generation, the library preparations were sequenced on an Illumina Novaseq platform, and 150 bp paired-end reads were generated.

### 2.3. Bioinformatic Analyses

Raw data (raw reads) in the fastq format were firstly processed through fastp software. In this step, clean data (clean reads) were obtained by removing reads containing adapters, reads containing 1 ploy-N and low-quality reads from raw data. Moreover, the Q_20_, Q_30_ and GC content of the clean data were calculated. All the downstream analyses were based on clean data with high quality.

The reference genome and gene model annotation files were downloaded from the genome website directly. The index of the reference genome was built using Hisat2 v2.0.5, and paired-end clean reads were aligned to the reference genome using Hisat2 v2.0.5. We selected Hisat2 as the mapping tool because Hisat2 can generate a database of splice junctions based on the gene model annotation file and thus a better mapping result than those of other non-splice mapping tools. 

The mapped reads of each sample were assembled via StringTie using a reference-based approach [34,35]. StringTie uses a novel network flow algorithm as well as an optional de novo assembly step to assemble and quantify full-length transcripts representing multiple splice variants for each gene locus. 

Counts v1.5.0-p3 was used to count the read numbers mapped to each gene. Then, the FPKM of each gene was calculated based on the length of the gene and the read count mapped to each gene. The FPKM, the expected number of Fragments Per Kilobase of transcript sequence per million base pairs sequenced, considers the effect of the sequencing depth and gene length for the read count at the same time and is currently the most commonly used method for estimating gene expression levels.

The differential expression analysis of two conditions/groups (two biological replicates per condition) was performed using the DESeq2 R package. DESeq2 provides statistical routines for determining differential expression in digital gene expression data using a model based on the negative binomial distribution. The resulting *p*-values were adjusted using Benjamini and Hochberg’s approach for controlling the false discovery rate. Genes with an adjusted *p*-value ≤ 0.05 found via DESeq2 were assigned as differentially expressed.

The Kyoto Encyclopedia of Genes and Genomes (KEGG) is a database resource used for understanding the high-level functions and utilities of the biological system, such as the cell, the organism and the ecosystem, from molecular-level information, especially large-scale molecular datasets generated by genome sequencing and other high-throughput experimental technologies (http://www.genome.jp/kegg/) (accessed on 25 December 2022) [36]. We used the cluster Profiler R package to test the statistical enrichment of differentially expressed genes in KEGG pathways.

### 2.4. Statistical Analysis

The summary statistics for data on the levels of chlorophyll, leaf water content and nitrogen in the leaves are shown as the mean and standard deviation for each day using the results from the four independent experiments. The Wilcoxon rank-sum test was used to determine the mean differences in levels across different groups because the dataset has a non-normal distribution based on the assessment of the Shapiro–Wilk test.We performed a post hoctest using the Bonferroni methodology to adjust *p*-values for pairwise comparisons. Significant differences were considered at an adjusted *p*-value of <0.05. All statistical analyses were carried out using SPSS V26.0.

## 3. Results and Discussion

### 3.1. Variations in the Physiological Parameters of Leaves

**Figure 1:**
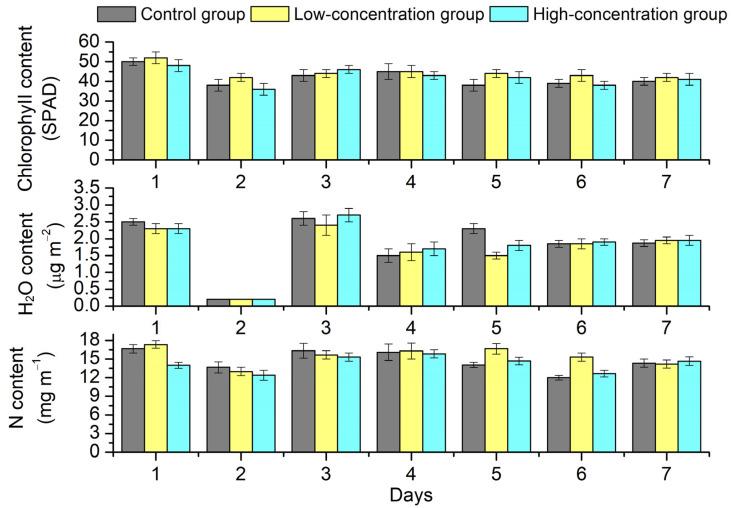
Effects of PAH stress on the mean levels of chlorophyll value (SPAD), water content (H_2_O) and nitrogen element (N) in the leaves.

Figure 1 presents the variations in the chlorophyll value, water content and nitrogen in the leaves in three groups during the experiment. For the control and exposure groups, the mean levels of chlorophyll, water content and nitrogen showed a similar variation trend, which decreased in the first two days and gradually increased on the third and fourth days due to watering. Then, the mean levels of chlorophyll, water content and nitrogen lowered steadily to the normal levels in the leaves. This finding indicates that humidity had an essential role in promoting the levels of chlorophyll and nitrogen in the leaves [37]. Watering on the leaves had a one-day delay effect on the variations in the levels of chlorophyll and nitrogen in the leaves. There were no significant differences in the levels of chlorophyll, water content and nitrogen across the low-concentration exposure group, high-concentration exposure group and control group. It is worth noting that the mean levels of chlorophyll in the low-exposure group were observed to be slightly higher than those in the control group, which may be attributable to the hormesis effects from PAHs [38]. In comparison, the mean levels of chlorophyll were mildly lower than those of the control group, which may be ascribed to the inhibition effects of high levels of PAHs [38].

### 3.2. Transcriptome Statistics

After the original data filtering, sequencing error check and the GC content distribution check, the clean reads were obtained for a follow-up subsequent analysis, and the data are summarized in the table below. As shown in Table 1, the levels of clean reads varied from 38.5 to 46.8 Gb, whichwere obtained from the sequencing library, with Q_30_ values in the range of 91.0–93.2% [39].

### 3.3. Differentially Expressed Genes

**Figure 2:**
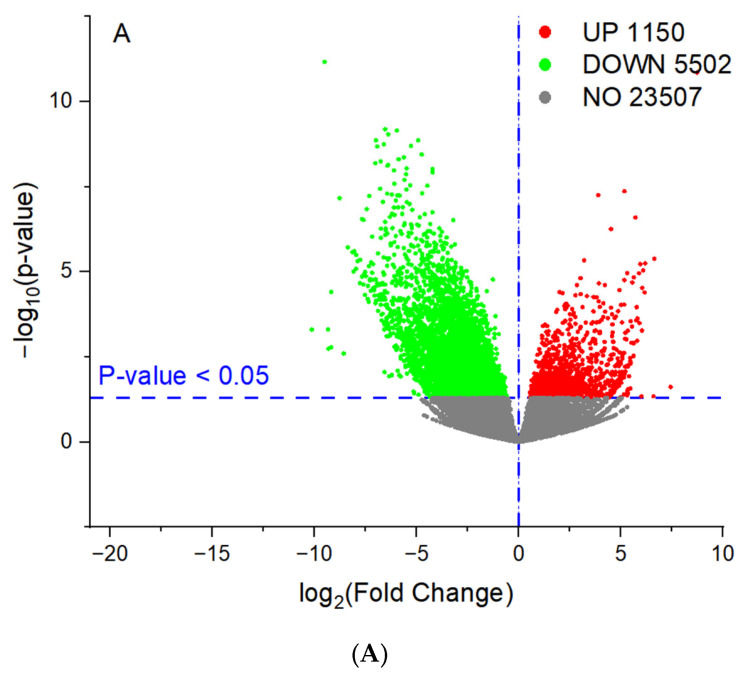

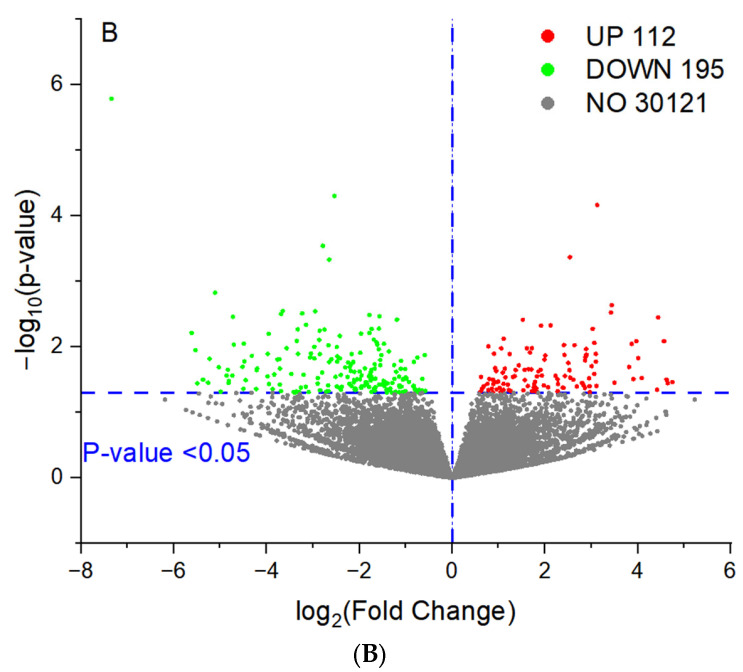
Number of upregulated and downregulated genes for high-concentration exposure group relative to the control (**A**) and low-concentration exposure group relative to the control (**B**).

We used the concentrations of individual PAHs at 5 μg L^−1^ to investigate the responses of leaves under exposure to PAHs at a high concentration. As shown in Figure 2A, significant differences in expressed genes were observed after 7 days of exposure between the high-concentration exposure group and the control group. The numbers of upregulated and downregulated genes of the high-concentration exposure group relative to the control group were 1150 and 5502, respectively. It is noteworthy that the concentrations of individual PAHs in the exposure group were much higher than the levels of individual PAHs at the environmentally relevant concentration in this study [23,24].

After exposure to individual PAHs at 0.1 μg L^−1^ for seven days, differences in expressed genes between the low-concentration exposure group and the control group were found to be insignificant. The numbers of upregulated and downregulated genes were 112 and 195, respectively. Although several studies have reported that exposure to PAHs could lead to changes in the physiological and biochemical processes of leaves, our results indicate that the changes in the expression of genes in leaves resulting from exposure to individual PAHs at environmentally relevant concentrations were insignificant. 

### 3.4. KEGG Enrichment Analysis

**Figure 3:**
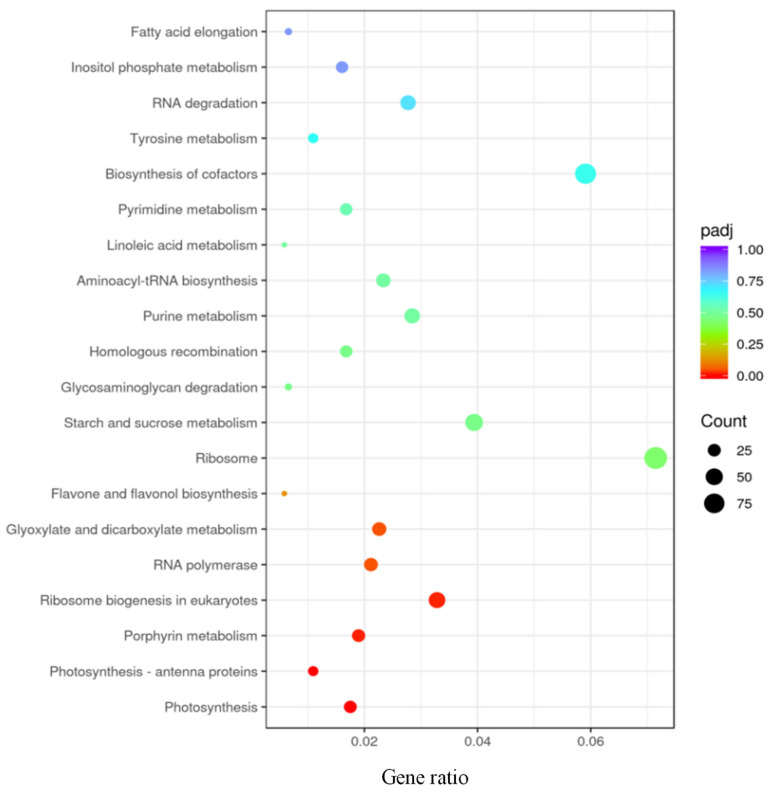
KEGG enrichment analysis of high-concentration exposure group relative to the control group. Scatter plot of KEGG enrichment, showing all pathways significantly enriched (*p* < 0.05) in each module.

Subsequently, we performed KEGG enrichment analysis to showwhich genes or pathways that the differentially expressed gene cluster was in. KEGGis a comprehensive database that integrates genome, chemistry and system function information [34]. A padj of less than 0.05 was used as the threshold for significant enrichment.For the comparison between the high-concentration exposure and the control group, significant differentially expressed genes were assigned into seven main pathways, which included flavone and flavonol biosynthesis, glyoxylate and dicarboxylate metabolism, RNA polymerase, ribosome biogenesis in eukaryotes, porphyrin metabolism, photosynthesis-antenna proteins and photosynthesis (Figure 3). The photosynthesis pathway includes 24 downregulated genes, and the photosynthesis-antenna proteins pathway has 15 downregulated genes. Moreover, the porphyrin metabolismpathway has 1 upregulated and 25 downregulated genes. Ribosome biogenesis in the eukaryotespathway has 2 upregulated and 43 downregulated genes. The RNA polymerasepathway has 3 upregulated and 26 downregulated genes. The glyoxylate and dicarboxylate metabolismpathway has 4 upregulated and 27 downregulated genes.

**Figure 4:**
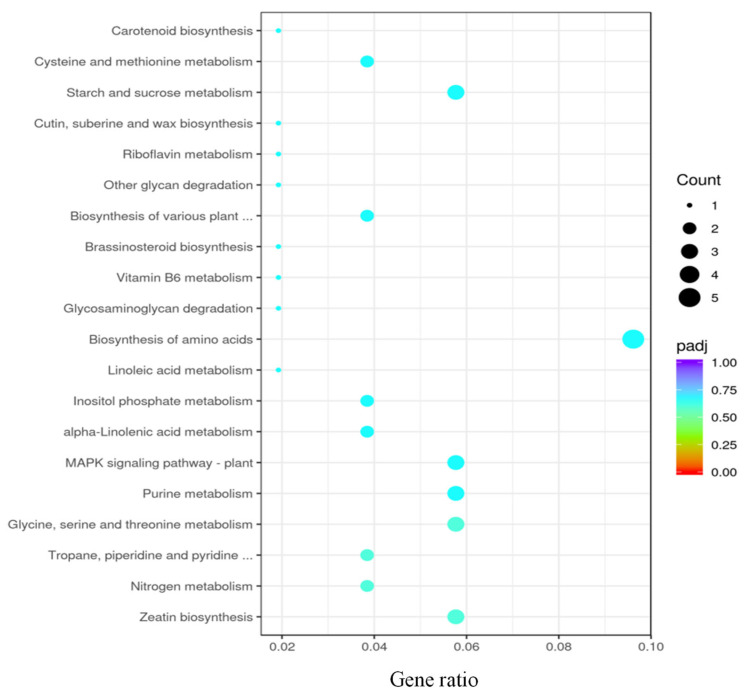
KEGG enrichment analysis of low-concentration exposure group relative to the control group. Scatter plot of KEGG enrichment, showing all pathways significantly enriched (*p* < 0.05) in each module.

In contrast, there were no significant differentially expressed genes in the KEGG enrichment analysis between the low-concentration exposure group and the control group (Figure 4). In general, the gene transcription levels obtained from the KEGG enrichment analysis exhibited a similar pattern and degree of alterations in comparison to the physiological results, thereby demonstrating that the responses of leaves to exposure to PAHs at environmentally relevant concentrations were negligible. 

In this study, the mixtures of 16 PAHs at environmental levels, not individual PAHs with high toxicity, were used to investigate the toxicological effects on the gene transcription levels of the leaves of *Rosa chinensis Jacq.* because these 16 types of PAHs have been frequently detected in the ambient environment [2,11,12,13,14,15,16]. Because individual PAHs with low toxicity could not influence the toxicity of other PAH species [1,7], the findings of this study could reflect the physiological conditions of leaves under exposure to PAHs at environmental levels in real situations. Future studies may focus on the physiological conditions of leaves under exposure to individual PAHs at environmental levels, which could aid in understanding the metabolism mechanism of leaves under exposure to individual PAHs with different toxicity. 

## 4. Conclusions

In this study, we measured the physiological parameters and gene transcription levels of the leaves of *Rosa chinensis Jacq.* exposedtoPAHsin the environmental concentration exposure group (0.1 μg L^−1^) and high-concentration exposure group (5 μg L^−1^). After exposure toPAHs at 5 μg L^−1^, significant differences in the gene transcription levels of the leaves of *Rosa chinensis Jacq.* were observed relative to the control group. These significant differentially expressed genes pertain to seven main pathways, which are flavone and flavonol biosynthesis, glyoxylate and dicarboxylate metabolism, RNA polymerase, ribosome biogenesis in eukaryotes, porphyrin metabolism, photosynthesis-antenna proteins and photosynthesis. On the contrary, environmentally relevantconcentrations of PAHs had no significant effect on the physiological parameters and gene transcription levels of the leaves of *Rosa chinensis Jacq.* during the 7-day exposure period in this study. Our results illustrate that exposure to ambient PAHs may havea negligible impact on the leaves of terrestrial plants at gene transcription levels. The findings of our study highlight the important roles of terrestrial plants in regulating ambient PAHs.

## Figures and Tables

**Table 1:** Summary of sample sequencing data quality.

Sample	Library	Raw Reads	Raw Bases	Clean Reads	Clean Bases	Error Rate	Q_20_	Q_30_	GCpct
C1	FRAS220304683-1r	45249234	6.79 G	44431476	6.66 G	0.03	97.42	92.76	45.9
C2	FRAS220304686-1r	47878640	7.18 G	46845610	7.03 G	0.03	97.36	92.7	48.22
C3	FRAS220304684-1r	44127062	6.62 G	42798502	6.42 G	0.03	97.03	91.98	48.1
C4	FRAS220304680-1r	40273130	6.04 G	39445930	5.92 G	0.03	97.49	92.9	45.73
L1	FRAS220304681-1r	42791504	6.42 G	42058236	6.31 G	0.03	97.54	93.02	45.14
L2	FRAS220304682-1r	46399720	6.96 G	45127820	6.77 G	0.03	97.33	92.69	47.96
L3	FRAS220304685-1r	45209526	6.78 G	43244788	6.49 G	0.03	97.03	92.12	48.26
L4	FRAS220304687-1r	45394486	6.81 G	44053436	6.61 G	0.03	97.4	92.76	47.11
H1	FRAS220304677-1r	46123300	6.92 G	44909222	6.74 G	0.03	97.18	92.4	48.88
H2	FRAS220304679-1r	39183628	5.88 G	38560642	5.78 G	0.03	97.44	92.72	45.4
H3	FRAS220304676-1r	42299468	6.34 G	41356972	6.2 G	0.03	97.28	92.45	45.95
H4	FRAS220304678-1r	39450472	5.92 G	38546398	5.78 G	0.03	96.65	91.03	45.57

Raw reads: Number of reads in the original data. Raw bases: Number of bases of the raw data (raw base = raw reads × 150 bp). Clean reads: Number of reads filtered from raw data. Clean bases: Number of bases filtered from the raw data (clean base = clean reads × 150 bp). Error rate: Overall data sequencing error rate. Q_20_: Percentage of bases with a Phred value greater than 20 in the total. Q_30_: Percentage of bases with a Phred value greater than 30 in the total. GCpct: Percentages of G and C in the four bases in clean reads.

## Data Availability

Data will be made available upon request from the corresponding author.

## References

[1] Beriro D.J., Cave M.R., Wragg J., Thomas R., Wills G., Evans F. (2016). A review of the current state of the art of physiologically-based tests for measuring human dermal in vitro bioavailability of polycyclic aromatic hydrocarbons (PAH) in soil. J. Hazard. Mater..

[2] Agus B.A.P., Rajentran K., Selamat J., Lestari S.D., Umar N.B., Hussain N. (2023). Determination of 16 EPA PAHs in food using gas and liquid chromatography. J. Food Compos. Anal..

[3] Wang X., Meyer C.P., Reisen F., Keywood M., Thai P.K., Hawker D.W., Powell J., Mueller J.F. (2017). Emission Factors for Selected Semivolatile Organic Chemicals from Burning of Tropical Biomass Fuels and Estimation of Annual Australian Emissions. Environ. Sci. Technol..

[4] Huang W., Huang B., Bi X., Lin Q., Liu M., Ren Z., Zhang G., Wang X., Sheng G., Fu J. (2014). Emission of PAHs, NPAHs and OPAHs from residential honeycomb coal briquette combustion. Energy Fuels.

[5] Shen G., Tao S., Wei S., Chen Y., Zhang Y., Shen H., Huang Y., Zhu D., Yuan C., Wang H. (2013). Field Measurement of Emission Factors of PM, EC, OC, Parent, Nitro-, and Oxy- Polycyclic Aromatic Hydrocarbons for Residential Briquette, Coal Cake, and Wood in Rural Shanxi, China. Environ. Sci. Technol..

[6] Liu W., Xu Y., Zhao Y., Liu Q., Yu S., Liu Y., Wang X., Liu Y., Tao S., Liu W. (2019). Occurrence, source, and risk assessment of atmospheric parent polycyclic aromatic hydrocarbons in the coastal cities of the Bohai and Yellow Seas, China. Environ. Pollut..

[7] Liu W., Wang J., Li W., Lin N., Liu Q., Xu M., Du W. (2022). Unexpected increase of PAH toxicity in ambient particulate matter under the implementation of clean air action: Evidence from two megacities in northern China. Air Qual. Atmos. Health.

[8] Shen G., Du W., Zhuo S., Yu J., Tao S. (2019). Improving regulations on residential emissions and non-criteria hazardous contaminants—Insights from a field campaign on ambient PM and PAHs in North China Plain. Environ. Sci. Policy.

[9] Chen J., Song Y., Liu Y., Chen W., Cen Y., You M., Yang G. (2023). DBP and BaP co-exposure induces kidney injury via promoting pyroptosis of renal tubular epithelial cells in rats. Chemosphere.

[10] Song S., Chen B., Huang T., Ma S., Liu L., Luo J., Shen H., Wang J., Guo L., Wu M. (2023). Assessing the contribution of global wildfire biomass burning to BaP contamination in the Arctic. Environ. Sci. Ecotechnol..

[11] Udom G.J., Frazzoli C., Ekhator O.C., Onyena A.P., Bocca B., Orisakwe O.E. (2023). Pervasiveness, bioaccumulation and subduing environmental health challenges posed by polycyclic aromatic hydrocarbons (PAHs): A systematic review to settle a one health strategy in Niger Delta, Nigeria. Environ. Res..

[12] Klingberg J., Strandberg B., Sjöman H., Taube M., Wallin G., Pleijel H. (2022). Polycyclic aromatic hydrocarbon (PAH) accumulation in Quercus palustris and Pinus nigra in the urban landscape of Gothenburg, Sweden. Sci. Total Environ..

[13] Yang M., Tian S., Liu Q., Yang Z., Yang Y., Shao P., Liu Y. (2022). Determination of 31 Polycyclic Aromatic Hydrocarbons in Plant Leaves Using Internal Standard Method with Ultrasonic Extraction-Gas Chromatography-Mass Spectrometry. Toxics.

[14] Sari M.F., Esen F., Tasdemir Y. (2021). Characterization, source apportionment, air/plant partitioning and cancer risk assessment of atmospheric PAHs measured with tree components and passive air sampler. Environ. Res..

[15] Wang Y., Zhang Z., Xu Y., Rodgers T.F.M., Ablimit M., Li J., Tan F. (2023). Identifying the contributions of root and foliage gaseous/particle uptakes to indoor plants for phthalates, OPFRs and PAHs. Sci. Total Environ..

[16] Giráldez P., Aboal J.R., Fernández J.Á., Di Guardo A., Terzaghi E. (2022). Plant-air partition coefficients for thirteen urban conifer tree species: Estimating the best gas and particulate matter associated PAH removers. Environ. Pollut..

[17] Nowak D.J., Ellis A., Greenfield E.J. (2022). The disparity in tree cover and ecosystem service values among redlining classes in the United States. Landsc. Urban Plan..

[18] Nowak D.J., Hirabayashi S., Bodine A., Greenfield E. (2014). Tree and forest effects on air quality and human health in the United States. Environ. Pollut..

[19] Oksanen E., Kontunen-Soppela S. (2021). Plants have different strategies to defend against air pollutants. Curr. Opin. Environ. Sci. Health.

[20] Han D., Shen H., Duan W., Chen L. (2020). A review on particulate matter removal capacity by urban forests at different scales. Urban For. Urban Green..

[21] Corada K., Woodward H., Alaraj H., Collins C.M., de Nazelle A. (2021). A systematic review of the leaf traits considered to contribute to removal of airborne particulate matter pollution in urban areas. Environ. Pollut..

[22] Yang B., Liu S., Liu Y., Li X., Lin X., Liu M., Liu X. (2017). PAHs uptake and translocation in Cinnamomum camphora leaves from Shanghai, China. Sci. Total Environ..

[23] Fellet G., Pošćić F., Licen S., Marchiol L., Musetti R., Tolloi A., Barbieri P., Zerbi G. (2016). PAHs accumulation on leaves of six evergreen urban shrubs: A field experiment. Atmos. Pollut. Res..

[24] Tian L., Yin S., Ma Y., Kang H., Zhang X., Tan H., Meng H., Liu C. (2019). Impact factor assessment of the uptake and accumulation of polycyclic aromatic hydrocarbons by plant leaves: Morphological characteristics have the greatest impact. Sci. Total Environ..

[25] Prigioniero A., Zuzolo D., Niinemets Ü., Postiglione A., Mercurio M., Izzo F., Trifuoggi M., Toscanesi M., Scarano P., Tartaglia M. (2022). Particulate matter and polycyclic aromatic hydrocarbon uptake in relation to leaf surface functional traits in Mediterranean evergreens: Potentials for air phytoremediation. J. Hazard. Mater..

[26] Yli-Pelkonen V., Viippola V., Rantalainen A.-L., Zheng J., Setälä H. (2018). The impact of urban trees on concentrations of PAHs and other gaseous air pollutants in Yanji, northeast China. Atmos. Environ..

[27] De Nicola F., Concha Graña E., López Mahía P., Muniategui Lorenzo S., Prada Rodríguez D., Retuerto R., Carballeira A., Aboal J.R., Fernández J.Á. (2017). Evergreen or deciduous trees for capturing PAHs from ambient air? A case study. Environ. Pollut..

[28] Anna K.-I., Emanuel G., Anna S.-R., Błońska E., Lasota J., Łagan S. (2018). Linking the contents of hydrophobic PAHs with the canopy water storage capacity of coniferous trees. Environ. Pollut..

[29] Nemecek-Marshall M., MacDonald R.C., Franzen J.J., Wojciechowski C.L., Fall R. (1995). Methanol Emission from Leaves (Enzymatic Detection of Gas-Phase Methanol and Relation of Methanol Fluxes to Stomatal Conductance and Leaf Development). Plant Physiol..

[30] Tao M., Xu Y., Liu Q., Liu Y., Tian S., Schauer J.J. (2023). Penetration of submicron amino-functionalized graphene quantum dots in plant stomata, implication for the depollution of atmospheric soot particles. Environ. Chem. Lett..

[31] Sabaratnam S., Gupta G., Mulchi C. (1988). Effects of nitrogen dioxide on leaf chlorophyll and nitrogen content of soybean. Environ. Pollut..

[32] Brown L.A., Williams O., Dash J. (2022). Calibration and characterisation of four chlorophyll meters and transmittance spectroscopy for non-destructive estimation of forest leaf chlorophyll concentration. Agric. For. Meteorol..

[33] Terzaghi E., De Nicola F., Cerabolini B.E.L., Posada-Baquero R., Ortega-Calvo J.-J., Di Guardo A. (2020). Role of photo- and biodegradation of two PAHs on leaves: Modelling the impact on air quality ecosystem services provided by urban trees. Sci. Total Environ..

[34] Sangwan R.S., Tripathi S., Singh J., Narnoliya L.K., Sangwan N.S. (2013). De novo sequencing and assembly of Centella asiatica leaf transcriptome for mapping of structural, functional and regulatory genes with special reference to secondary metabolism. Gene.

[35] He J., Fu T., Zhang L., Wanrong Gao L., Rensel M., Remage-Healey L., White S.A., Gedman G., Whitelegge J., Xiao X. (2022). Improved zebra finch brain transcriptome identifies novel proteins with sex differences. Gene.

[36] Huang F., Fu M., Li J., Chen L., Feng K., Huang T., Cai Y.-D. (2023). Analysis and prediction of protein stability based on interaction network, gene ontology, and KEGG pathway enrichment scores. Biochim. Biophys. Acta (BBA)-Proteins Proteom..

[37] Chen Y., Cai X., Tang B., Xie Q., Chen G., Chen X., Hu Z. (2023). SlERF.J2 reduces chlorophyll accumulation and inhibits chloroplast biogenesis and development in tomato leaves. Plant Sci..

[38] Calabrese E.J., Blain R.B. (2009). Hormesis and plant biology. Environ. Pollut..

[39] Zhou S., Yang Q., Song Y., Cheng B., Ai X. (2023). Effect of Copper Sulphate Exposure on the Oxidative Stress, Gill Transcriptome and External Microbiota of Yellow Catfish, Pelteobagrusfulvidraco. Antioxidants.

